# Anomaly Detection with Graph Embedding in Bioinformatics: A Survey

**DOI:** 10.2174/0113892029378251251210215239

**Published:** 2026-03-27

**Authors:** Tong Wang, Zhi-Ping Liu

**Affiliations:** 1 Department of Biomedical Engineering, School of Control Science and Engineering, Shandong University, Jinan, Shandong 250061, China

**Keywords:** Bioinformatics, graph embedding, anomaly detection, neural network, biomarker discovery, multi-omics data

## Abstract

With the rapid development of network and data sciences, graph anomaly detection has emerged as a key research direction across multiple domains, and its application has been widely recognized in bioinformatics. Biomolecular networks, including gene regulatory networks, protein-protein interaction networks, and drug-molecule interaction networks, form the foundation of complex biological processes within organisms. Abnormal patterns within these networks are often closely associated with the onset and progression of diseases, making graph anomaly detection critically important for revealing disease mechanisms, facilitating disease diagnosis, and advancing drug development. In this paper, we review the current status of graph embedding-based anomaly detection algorithms in bioinformatics, with a particular focus on key literature and the development of dynamic graph anomaly detection algorithms. We explore how graph embedding techniques learn high-dimensional features of biomolecular networks and represent them in a low-dimensional space. Furthermore, we investigate the dynamic nature of biomolecular networks and how graph embedding algorithms can capture these changes to improve detection accuracy and efficiency. The challenges of current methods are summarized, and future directions are proposed to support key applications such as disease diagnosis, gene function annotation, and drug discovery. Through this review, we aim to provide theoretical foundations and methodological support for network anomaly detection in high-throughput omics data.

## 1 INTRODUCTION

As network science becomes more widespread and the volume of data grows rapidly, graph anomaly detection has emerged as an important research area attracting significant attention. In various domains such as social networks, network intrusion detection [1], telecommunications fraud [2], healthcare monitoring [3], and financial transaction systems [4], graph anomaly detection plays a crucial role. Within this context, bioinformatics, as a critical interdisciplinary field, has also adopted graph anomaly detection methods in its diverse research areas.

In bioinformatics, molecules within cells do not function in isolation but exist in the form of signaling pathways or functional modules. Therefore, the relationships between molecules within cells can be viewed as a complex network [5]. For instance, gene regulatory networks and protein-protein interaction networks are large-scale graphs that represent these interactions. Gene regulation, a critical mechanism for controlling gene expression within organisms, along with gene relationships and expressions, can be represented graphically, where nodes represent genes and edges signify the regulatory relationships between them. Abnormal gene regulation or expression may be associated with the occurrence or development of diseases [6]. Networks of protein interactions can similarly be considered as graphs. Proteins, which are essential for various biological functions, can indicate underlying disease states or abnormalities in biological processes if their expression or interactions become disrupted [7]. Therefore, by applying graph anomaly detection algorithms, it is possible to identify abnormal genes, proteins, or their interactions in specific disease states, thereby uncovering potential biological mechanisms and providing clues for disease diagnosis and treatment.

Additionally, interactions between molecular compounds can also be structured into graphs. For example, networks of interactions between drug molecules and target proteins can aid in understanding drug mechanisms and side effects [8]. Graph anomaly detection algorithms can be utilized to identify molecules within drug interaction networks that exhibit unusual activities or interaction patterns, assisting in drug development and the assessment of drug safety. Thus, by modeling biomolecular networks, the complex structures and relationships between molecules can be represented as general graphs for anomaly detection, offering a powerful tool for identifying abnormalities within biomolecular networks.

**Fig. (1):**
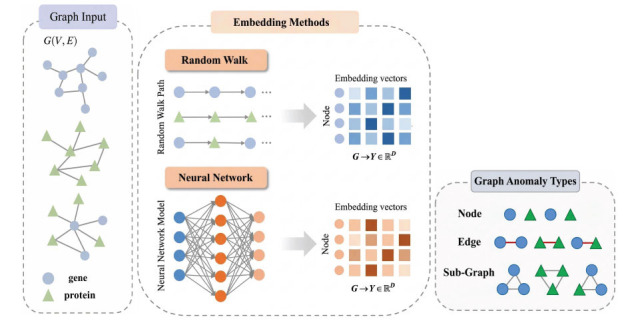
Overall framework of graph embedding for anomaly detection in biomolecular networks.

However, biomolecular networks are characterized by high data throughput, high dimensionality, and structural complexity [6]. These characteristics present significant challenges in the analysis and understanding of such networks. Therefore, when performing graph anomaly detection in biomolecular networks, effectively extracting patterns from data for accurate detection and prediction becomes a major challenge. Graph embedding algorithms provide a promising solution to these challenges [9]. As shown in Fig. **1**, graph embedding algorithms employ techniques such as random walks and graph neural networks to learn the high-dimensional features of nodes and edges within complex graphs and represent them in a lower-dimensional space. This process facilitates the extraction and characterization of complex graph information and structures within biomolecular networks, enabling downstream tasks such as graph anomaly detection.

Moreover, the graphical information and structure within biomolecular networks often evolve over time, with edges and nodes appearing or disappearing as time progresses [10]. Consequently, dynamic graph anomaly detection algorithms based on graph embedding have attracted extensive attention from bioinformaticians due to their ability to effectively capture the evolving characteristics of graphs. This capability allows them to better meet practical needs within specific biomolecular networks.

The primary goal of this article is to review the current state of graph embedding-based anomaly detection algorithms in bioinformatics, with a particular focus on key literature concerning dynamic graph anomaly detection algorithms, and to emphasize their critical roles in accelerating biomedical data science. We focus specifically on biomolecular networks, including drug-related networks, gene regulatory networks, protein interaction networks, and multi-omics heterogeneous networks. By summarizing existing research methodologies and highlighting the challenges and future directions of graph embedding-based anomaly detection algorithms, this article aims to provide a robust knowledge base to support further research in disease diagnosis, gene function analysis, and drug discovery, among other areas in bioinformatics.

## 2 DEFINITION AND SYMBOLS

Currently, there is no unified definition for graph embedding-based anomaly detection in bioinformatics, as its interpretation often depends on the specific application or scenario. Therefore, this section will take gene regulatory networks in bioinformatics as an example. Starting from the definition of graphs in gene regulatory networks, static and dynamic graphs will be introduced, and the definition of graph anomalies will be summarized from the perspectives of gene nodes, edges, subgraphs, *etc*. Similarly, analogous definitions can be extended to graph-embedding-based anomaly detection in other bioinformatics applications.

### 2.1 Definition of Graph

As a general data structure, graphs are widely used to represent complex structured data. Compared with other data structures, they more effectively store and express entities and their relationships. In the real world, networks are widely used in social network analysis [11], Web network analysis [12], transportation network optimization [13], knowledge graph construction [14], and many other fields. In gene regulatory networks, the regulatory interactions and expression between genes can also be represented by graphs, where nodes represent genes and edges represent regulatory interactions between them.

#### 2.1.1 Static Graph

In a gene regulatory network, a static graph can be expressed as a collection of gene nodes and gene regulatory relationships, which can be represented as *G* = (*V, E*). Among them *V* = {*v*_1_, *v*_2_, *v*_3_, ..., *v*_n_} is the set of gene nodes in the graph, while *E* = {*e*_ij_}^n^_i,j=1_ is the set of gene regulatory relationships in the graph. *e*_ij_ represents the weight of the edges connecting the gene nodes *i* and *j* in the graph. In an unweighted network *e*_ij_ = 1. Suppose *e*_ij_ = 0 it indicates that there is no regulatory relationship between the two gene nodes. For a weighted network, the definition is similar, with only differences in the definition of edge weights. If there is a connection from gene node *i* to gene node *j* without a reverse edge, that is, gene node *i* has a regulatory relationship with gene node *j*, while gene node *j* has no regulatory relationship with gene node *i*, then there is an *e*_ij_ > 0 and *e*_ji_ = 0.

**Fig. (2):**
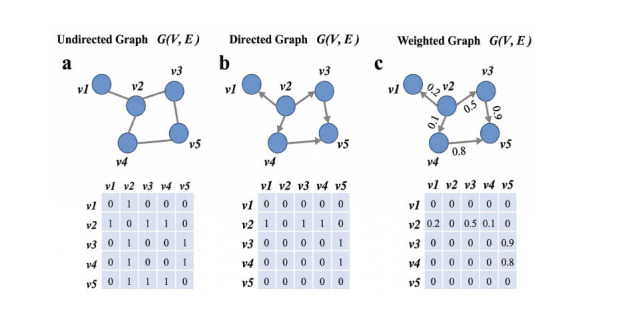
The undirected, directed, weighted graphs and their adjacency matrices.

In addition, an adjacency matrix is also a common representation of graphs. As shown in Fig. **2**, an adjacency matrix is a two-dimensional matrix, where rows and columns correspond to nodes in the gene regulatory network, and the matrix elements represent the regulatory relationships between gene nodes. For undirected graphs, the adjacency matrix is symmetric, while gene regulatory networks are generally directed graphs. Therefore, for gene regulatory networks, the adjacency matrix may not necessarily be symmetric.

#### 2.1.2 Dynamic Graph

**Fig. (3):**
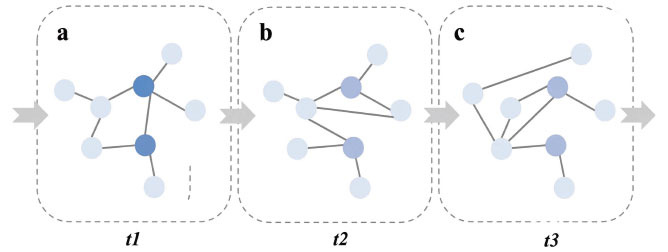
Dynamic graph evolution process of a gene regulatory network.

The dynamic graph refers to a graph in which the nodes and edges in the graph change over time, as illustrated in Fig. **3**. In the dynamic gene regulatory networks, operations such as adding, deleting, and modifying nodes and edges can capture the evolution process and structural changes of the gene regulatory network.

The dynamic graph of the gene regulatory network can be represented by *G* = (*V*, *E*, *T*), where *V* = {*v*_1_, *v*_2_, *v*_3_, ..., *v*_n_} is the set of gene nodes, *E* = *e*_1_, *e*_2_, *e*_3_, ... *e*_m_ is the set of edges, *T* = *t*_1_, *t*_2_, *t*_3_, ... *t*_m_ and is a set of timestamps that represent the creation time of gene nodes and edges in the gene regulatory network. The dynamic diagram of the gene regulatory network can be represented as *G* = (*V*, *E*, *T*, *τ*). Among them, *τ* is a mapping function that can map each gene node and edge to their state at different timestamps. From the perspective of graph snapshots, the dynamic graph of gene regulatory networks can be defined as a set of graph snapshot sequences. The dynamic graph can be represented as *G* = *G*_1_, *G*_2_, *G*_3_, ... *G*_n_. Among them, *G*_i_ = (*V*_i_, *E*_i_) represents the snapshot of the gene regulatory network graph at the *i* timestamp, that is, at timestamp *t*_i_, the set of gene nodes in the gene regulatory network is *V*_i_, and the set of edges is *E*_i_.

### 2.2 Definition of an Anomaly in Graph

Moonesinghe *et al.* [15] define anomaly detection as discovering target objects that are different from most objects. In gene regulatory networks, an anomaly in a graph typically refers to gene nodes, edges, or subgraphs that show significant deviations from observed patterns in the graph. Below, anomalies in the gene regulatory network graph are defined from three aspects: nodes, edges, and subgraphs.

#### 2.2.1 Node Anomaly

In gene regulatory networks, a node anomaly refers to gene nodes with characteristics significantly different from surrounding nodes. These gene nodes may exhibit abnormal expression patterns, atypical regulatory states, or irregular functional characteristics. Node anomalies can be considered genes with special biological importance or abnormal regulatory status in regulatory networks.

#### 2.2.2 Edge Anomaly

An edge anomaly refers to a regulatory relationship between two gene nodes that deviates significantly from expected patterns or normal biological processes. For example, certain genes may have abnormal co-expression or interaction relationships, or the regulatory relationships between certain genes may not be consistent with normal biological processes. Edge anomalies may indicate disruptions in important regulatory pathways or aberrant associations between essential genes in the gene regulatory network.

#### 2.2.3 Subgraph Anomaly

A subgraph anomaly refers to abnormal patterns or substructures within the gene regulatory network. These abnormal subgraphs may consist of a set of related gene nodes and their regulatory relationships, forming a significant subnetwork within the entire network. Such subgraphs may represent specific biological processes, regulatory pathways, or gene functional modules in certain disease states.

## 3 GRAPH EMBEDDING METHODS

The graph embedding method refers to mapping nodes or edges in a graph to a low-dimensional vector space, while preserving the topological structure and attribute information in the graph for further analysis and application [9]. This method can be regarded as a compression and transformation process, enabling the representation and processing of graph information using lower-dimensional vectors.

**Fig. (4):**
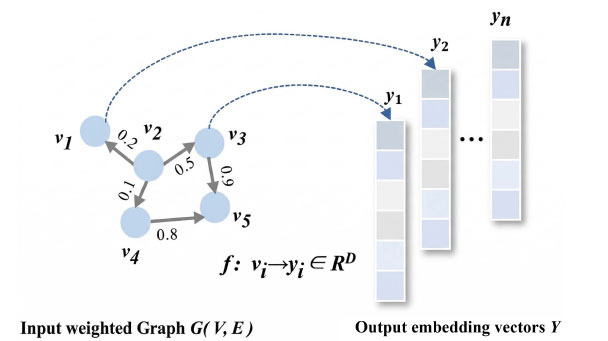
The schematic diagram of graph embedding methods.

Combining the previous definitions of graph in gene regulatory networks, given a graph *G* = (*V, E*), the graph embedding method maps nodes to a low-dimensional vector space using a mapping function 𝑓 : 𝑣_𝑖_ → 𝑦_𝑖_ ∈ 𝑅^𝐷^, where 𝐷 represents the dimension of the expected node vector. After embedding the graph, low-dimensional vector representations of each node in the network *G* are obtained, which is particularly suitable for large-scale biomolecular data (as shown in Fig. **4**).

In this section, we will introduce the most advanced graph embedding methods and classify them into two categories: graph embedding methods based on random walks and neural networks (as shown in Table **1**). Additionally, we provide an overview of the main literature related to the application of embedding technology of networks in bioinformatics (as shown in Table **2**).

### 3.1 Graph Embedding Method Based on Random Walks

In graph theory, the method based on random walks captures the structural relationships between nodes. By performing truncated random walks, the graph is transformed into a sequence of nodes, *i.e*., paths, thereby preserving the structural proximity of the graph.

In natural language processing models, the Word2vec model [16] learns from large amounts of text data and maps words to a continuous vector space, thereby capturing semantic relationships between words and enabling vocabulary representation and semantic inference. It includes Continuous Bag of Words (CBOW) and Skip-gram models, which predict target words based on context and predict contextual words based on target words, respectively, to achieve word vector learning.

Inspired by the Word2vec model, Perozzi *et al.* proposed the DeepWalk algorithm [17], which introduced deep learning techniques into the field of graph embedding. In this approach, a random walk is used to generate a sequence of nodes, which is similar to sentences in a document, where nodes are analogous to words in text. After training, the vector representation of the nodes is obtained, preserving the neighborhood structure of the nodes in the graph.

The Node2Vec algorithm [18] builds on DeepWalk and improves the generation of random walk paths by introducing two hyperparameters to control the depth and breadth of sampling. This algorithm effectively explores different neighborhoods through biased random walks. WalkLets [19] is another extension of DeepWalk that modifies the basic random walk strategy by skipping some nodes during each walk, thereby preserving global structural information.

Tang *et al.* proposed the Large-scale Information Network Embedding (LINE) algorithm [20], which models the probability of all first-order and second-order similarity nodes and minimizes the distance between the probability distribution and the empirical distribution to obtain the final vector representation. This algorithm efficiently handles large-scale networks while maintaining both local and global information of the network structure.

The GraphWave algorithm [21] learns from the graphical representation of quantified nodes through structural similarity features. It uses a thermal kernel wave graph to capture the neighborhood structure of nodes, effectively representing nodes in continuous space. This algorithm is suitable for capturing local structural features of nodes so that nodes with similar structures are mapped to similar positions in vector space.

The Hierarchical Representation Learning for Networks (HARP) algorithm proposed by Chen *et al.* [22] generates a node sequence through random walks and uses the learned node sequence to obtain hierarchical embeddings of nodes, maintaining high-order proximity between nodes. Additionally, similar to DeepWalk, Metapath2vec [23] formalizes meta-path-based random walks and introduces heterogeneous versions of the Skip-gram model to learn node embeddings.

### 3.2 Graph Embedding Method Based on Neural Networks

In recent years, neural network models have achieved significant success in many fields. Various neural networks have also been introduced into graph embedding, such as Graph Auto-Encoders (GAE) [24], Generative Adversarial Networks (GAN) [25], Graph Convolutional Networks (GCN) [26], and Graph Attention Networks (GAT) [27]. The application of neural networks in graph embedding effectively transforms complex graph-structured data into low-dimensional vector representations through the powerful capabilities of deep learning, thereby supporting various downstream machine learning tasks.

The GCN [26] proposed by Kipf *et al.* is a graph embedding method that learns node representations by performing convolution operations on graph-structured data. GCN is a semi-supervised learning framework that captures the local connection patterns of neighboring nodes by aggregating their features into the current node. The GAT [27] proposed by Veličković *et al.* introduces an attention mechanism, allowing the model to assign different weights to each neighbor when aggregating neighbor information. This mechanism enables GAT to capture relationships between nodes more flexibly, especially in handling the heterogeneity and complexity of graphs.

Hamilton proposed GraphSAGE [28], an inductive learning method that can learn a function from graph data to generate embeddings for unseen nodes. Unlike traditional graph embedding methods, GraphSAGE generates node embeddings by sampling and aggregating feature information from neighboring nodes, supporting effective learning of large-scale graphs.

The GAE [24] proposed by Kipf *et al.* uses unsupervised learning to learn graph embeddings. It typically consists of two parts: the encoder encodes nodes into low-dimensional vector representations, and the decoder reconstructs the original structure of the graph from these embeddings. GAEs are particularly suitable for graph data without node labels or edge weights, learning effective node representations by reconstructing the graph structure.

The Structural Deep Network Embedding (SDNE) algorithm [29] proposed by Wang *et al.* is a deep learning-based network representation model that uses autoencoders and local preservation constraints to learn node representations. The Transformer [30] utilizes self-attention mechanisms to capture long-range dependencies in graphs, enabling it to model both local and global relationships effectively. This approach enhances the model’s ability to handle complex graph data by focusing on different parts of the graph with varying levels of importance.

In the time complexity, *N* represents the number of nodes, *E* represents the number of edges, *d* represents the embedding dimension, *N*_m_ represents the number of meta path nodes in Metapath2vec, *S* and represents the number of sampled neighbours in GraphSAGE.

### 3.3 Applications in Bioinformatics

However, to date, these advanced graph embedding methods have primarily been evaluated on non-biomedical networks with only a limited number of studies providing evaluations and analyses for biomolecular networks, which have yet to be thoroughly explored. In the following sections, we will review the key literature related to the application of the aforementioned techniques in various omics data analyses and drug data analyses. Table **2** lists the papers mentioned in this review.

#### 3.3.1 Omics Data

Omics data includes genomes, proteomics, metabolomics, and so on. Molecular network research on omics data can identify abnormal molecules or their interactions in specific disease states, thereby revealing potential biological mechanisms and providing clues for disease diagnosis and treatment.

Suratanee *et al.* [32] used the DeepWalk algorithm to capture complex relationships between genes in autism spectrum disorder (ASD) and obtained gene embedding representations for subsequent identification of new ASD associations, contributing to ASD research and promoting the discovery of new targets for molecular targeted therapy. Zhu *et al.* [33] proposed a new graph-based machine learning framework to predict disease-related genes and also used the DeepWalk algorithm to obtain a low-dimensional vector representation of the graph embedding. Unlike the algorithm proposed by Suratanee *et al.*, this framework uses low-dimensional vectors for subsequent graph convolutional networks to learn gene-disease associations and shows strong performance in gene priority ranking.

Josifoski *et al.* [34] applied Node2Vec to the PPI network to embed proteins while preserving local and global topological structures, then used the embeddings to train a binary classifier for protein function prediction. Zitnik *et al.* proposed the OhmNet algorithm [35] for hierarchical-aware unsupervised node feature learning in multi-layer networks. This algorithm automatically learns the mapping of proteins represented by nodes to a low-dimensional feature space using neural networks by constructing a multi-layer network. It enables more accurate predictions of cellular function in multiple tissues with known tissue-specific functions and generates precise hypotheses about the roles of tissue-specific proteins.

Kang *et al.* proposed a novel Graph Neural Network (LR-GNN) [36] based on link representation to identify potential molecular associations and applied a GCN encoder to obtain node embeddings for constructing Link Representation (LR). It shows robust performance in biomedical network data such as lncRNA-disease associations, miRNA-disease associations, and protein-protein interactions. Lu *et al.* proposed a computational framework based on the metabolite-gene-disease heterogeneous network (MGDHGS) [37] to predict potential associations. Using GraphSAGE to learn low-dimensional neighborhood representations in metabolite-gene-disease heterogeneous networks supports the prediction of associations between metabolites and diseases, enhancing our understanding of complex metabolite-gene-disease interactions. Lagisetty *et al.* developed the GeneEMBED algorithm [38], which uses GraphWave to obtain embedded representations of molecular networks for evaluating disease gene associations. This algorithm identifies new candidate genes in two Alzheimer’s disease cohorts and three networks, demonstrating its wide applicability in complex diseases.

#### 3.3.2 Drug Data

Research on drug data networks includes the prediction of drug-target interactions and drug-disease interactions, aiming to identify molecules with abnormal activity or interaction patterns, thereby supporting drug development and safety assessment.

Ma *et al.* [39] integrated drug similarity using a multi-view graph autoencoder with attention mechanisms, enhancing drug interaction prediction by weighting different drug views. Chen *et al.* [40] developed a DeepWalk-based embedding method for predicting drug-target interactions from multiple molecular networks. Experimental results indicate that behavioral features perform better across classifiers, especially random forest classifiers. This approach is suitable for predicting drug-target interactions (DTI) and offers a new perspective for predicting associations with other biomolecules.

Purkayastha *et al.* [41] proposed a method for predicting drug interactions using rich drug representations from multiple knowledge sources. They used the drug-target interaction network to learn drug graph embeddings through Metapath2vec and applied a link prediction algorithm based on graph autoencoders to predict additional edges as potential interactions, achieving strong performance on three benchmark drug datasets. Hu *et al.* [42] proposed an end-to-end neural network model that embeds protein structure and drug features into a graph through a GAT and evaluates interactions *via* a fully connected layer, demonstrating strong results on the PDBBind dataset.

Liu *et al.* [43] proposed a drug interaction prediction framework using deep attention neural networks to predict unobserved drug interactions. This method uses SDNE graph embedding to learn drug representations from drug interaction networks for accurate future predictions. Feng *et al.* [44] proposed a deep learning framework based on GCN, which obtains low-dimensional vector representations of drug-disease interaction networks for subsequent link prediction. This method demonstrates the superiority of GCN-derived low-dimensional vectors in predicting potential drug-disease effects compared to other models.

## 4 ANOMALY DETECTION METHODS

In the previous section, we discussed the research status and progress of graph embedding methods, as well as their application performance in bioinformatics. These methods effectively map graph information and structure in bioinformatics to a low-dimensional space, retaining the structural characteristics of the original graph and providing strong support for downstream learning tasks. In this section, we summarize four types of anomaly detection methods based on graph embedding and review key literature (as shown in Table **3**).

### 4.1 Applications in Bioinformatics

The number of anomaly detection methods based on graph embedding is considerable and can be roughly divided into four categories:

#### 4.1.1 Based on Distance

A simple and intuitive approach measures the similarity between nodes using the distance of their graph embedding representations. Abnormal nodes are typically distinct from other nodes due to unique characteristics. The distance between each node and its neighbors can be calculated and compared with the average distance of other nodes. Nodes with larger distances may be considered anomalies.

#### 4.1.2 Based on Density

Anomaly detection can also be performed based on the local density represented by graph embeddings. Abnormal nodes are often located in sparse regions with lower surrounding node density. Density estimation techniques, such as K-nearest neighbor methods or local outlier factors, can calculate the local density of nodes and identify nodes with low density as anomalies.

#### 4.1.3 Based on Graph Structure

When embedding representations are learned from graph structure, the topology of the graph can be leveraged for anomaly detection. This includes information such as neighboring nodes, their connection patterns, and the positions of nodes in the graph. Graph-based methods may use topological features such as node degree, clustering coefficient, betweenness centrality, and others.

#### 4.1.4 Based on Probability Models

If the graph embedding representation is considered a sample from the data distribution, a probability model can be used to identify anomalies. For example, Gaussian Mixture Models (GMM) or other probability distributions can fit the embedded representations, with nodes having lower probabilities in the model considered anomalies.

The four types of methods outlined above are common approaches to anomaly detection. Their practical application in bioinformatics requires selecting or designing suitable methods based on the specific context. Additionally, experimental validation is usually needed to evaluate the performance of different methods, and tuning and selection are guided by experimental results.

### 4.2 Overview of Anomaly Detection Methods Based on Graph Embedding

Anomaly detection aims to identify data points that do not conform to expected patterns, which is particularly important in bioinformatics because it can help detect abnormal biological signals that may indicate diseases, genetic variations, or other physiological anomalies. Bioinformatics data are typically high-dimensional, complex, and contain substantial noise, making traditional anomaly detection methods difficult to apply directly. Below, we review the relevant literature on graph embedding-based anomaly detection methods applicable in bioinformatics. Table **3** lists the papers discussed in this section.

Ding *et al.* [45] used GCN for node embedding learning and employed deep autoencoders to reconstruct the original data from the learned embeddings. By combining GCN and autoencoders, the reconstruction error of nodes was measured from both graph structure and attribute perspectives to detect anomalies.

Li *et al.* [46] proposed the SpecAE model, which uses a graph convolutional encoder to learn node representations and reconstructs node attributes through a deconvolution decoder. Density estimation and Gaussian Mixture Models (GMM) were used to detect global anomalies and community anomalies, respectively, and the model’s effectiveness was verified on real data.

Fan *et al.* [47] proposed the AnomalyDAE algorithm, which achieves anomaly detection based on graph embedding. Graph attention networks were used to encode graph structure information, and a separate attribute autoencoder was used to embed node attributes. Through an unsupervised encoding-decoding process, nodes are ranked based on reconstruction loss, with the top-ranked nodes identified as outliers.

The DONE algorithm [48] employs two independent autoencoders, trained to minimize reconstruction errors while maintaining homomorphism. The anomaly score for each node is quantified by minimizing the sum of loss functions, and nodes with the highest scores are selected as anomalies.

Considering the presence of anomalous links in graphs, Duan *et al.* [49] proposed the Anomaly Aware Network Embedding (AANE) model, which generates node embeddings through GCN for anomaly prediction. Edges with anomaly probabilities below a defined threshold are recognized as anomalies, and the k edges with the lowest probabilities are selected as anomalous.

## 5 DYNAMIC GRAPH ANOMALY DETECTION BASED ON GRAPH EMBEDDING

Although the application of graph embedding for anomaly detection has achieved promising results in bioinformatics, it still faces some unresolved challenges. We particularly emphasize the following key issues:

Due to the complex interactions in biomolecular networks, network structure and node interactions may change under different physiological conditions, disease states, or developmental stages. Dynamic graph anomaly detection methods based on graph embedding can handle this variability by comparing graph embeddings at different time points to identify abnormal changes. This is crucial for understanding disease mechanisms, discovering biomarkers, and related applications. Therefore, dynamic graph anomaly detection based on graph embedding is highly significant in bioinformatics. Below, we review the development and research status of the most commonly used dynamic graph anomaly detection algorithms based on graph embedding. The relevant papers are listed in Table **4**.

Dynamic graph anomaly detection involves identifying abnormal behavior in time-series data, and graph embedding-based methods have achieved notable results in this area. Yu *et al.* [50] proposed the NetWalk algorithm, which was the first to apply network embedding to dynamic network anomaly detection, representing a pioneering innovation. NetWalk uses a deep autoencoder to learn vector representations through network walks and dynamically updates node embeddings *via* a reservoir sampling algorithm, enabling accurate and real-time anomaly detection of continuously evolving networks. However, NetWalk updates only edge representations and does not capture long-term and short-term evolution patterns of nodes and graph structures.

To address this limitation, Zheng *et al.* [51] proposed the AddGraph model, which combines GCN and GRU (Gated Recurrent Unit) to integrate temporal, structural, and attribute information, capturing more representative structural features from the dependency relationships in temporal graphs. Considering that NetWalk and similar methods first learn node embeddings and then perform anomaly detection—without end-to-end training—Cai *et al.* [52] proposed StrGNN, an end-to-end structured dynamic graph neural network model for detecting abnormal edges in dynamic graphs.

Since both AddGraph and StrGNN use separate modules to extract spatial and temporal features, their ability to capture coupled spatiotemporal information is limited. To overcome this, Liu *et al.* [53] proposed TADDY, a transformer-based dynamic network anomaly detection framework. TADDY constructs comprehensive node encodings to better represent structural and temporal information and captures spatiotemporal patterns through a dynamic network Transformer model.

With continued research, many effective algorithms have been developed for different applications. Ding *et al.* [54] proposed Graph Deviation Network (GDN), a GNN-based structural learning model for multivariate time-series anomaly detection. The model also uses attention weights learned by GAT to explain detected anomalies, accurately identifying anomalies while capturing sensor relationships to help locate root causes. Yang *et al.* [55] proposed a stochastic neural network, H-VGRAE (Hierarchical Variational Graph Recurrent Autoencoder), to detect anomalies in dynamic graphs, leveraging high flexibility and randomness by modeling nodes as random variables. Zhang *et al.* [56] applied node anomaly detection to software API call links and proposed the Dynamic Evolutionary Graph Convolutional Network (DEGCN). Wang *et al.* [57] developed a Hierarchical Graph Convolutional Network (HGCN) for edge detection in dynamic graphs, integrating graph neural networks with recurrent neural networks. Wan *et al.* [58] proposed GLAD-PAW, a graph-based logarithmic anomaly detection algorithm that uses a position-aware weighted graph attention layer and global attention readout to learn embeddings of records and session graphs for subsequent anomaly detection.

To evaluate these models, we conducted performance tests on several algorithms using the UCI dataset. We injected anomalous data into the test set at three different proportions: pA = 1%, 5%, and 10%. The AUC results indicate that the transformer-based anomaly detection algorithm outperforms the others. This superior performance can be attributed to its strong capability in capturing long-range dependencies and modelling complex relationships within the data.

## 6 CHALLENGES OF ANOMALY DETECTION BASED ON GRAPH EMBEDDING IN THE FIELD OF BIOINFORMATICS

Although the use of graph embedding for anomaly detection has achieved promising results in bioinformatics, several unresolved challenges remain. We particularly emphasize the following key issues:

### 6.1 Preserving Structural and High-Dimensional Information

Due to the high structural complexity and dimensionality of biomolecular networks, they cannot be directly analyzed in their raw form. Although graph embedding methods map these networks to a low-dimensional space for analysis, it is not guaranteed that all graph information is preserved. Maintaining the integrity of graph information is crucial for ensuring the accuracy of subsequent analyses [59]. Therefore, ensuring that as much information as possible is preserved during graph embedding is a primary challenge that needs to be addressed.

### 6.2 Challenge of Interpretability in Graph Embedding-Based Anomaly Detection for Biomedical Omics Data

Most graph embedding-based anomaly detection algorithms are not specifically designed for high-throughput biomedical omics data, and no unified framework exists for selecting the most appropriate methods. A key challenge is ensuring that these techniques not only adapt to the unique characteristics of omics data but also maintain model interpretability. In bioinformatics, interpretability is essential for validating biological findings and guiding clinical decisions [60]. However, many deep learning-based graph embedding models operate as “black boxes,” making it difficult to understand how anomalies are identified. This lack of transparency reduces trust in model predictions and limits their application in critical areas such as precision medicine and biomarker discovery, where explainable decision-making is imperative.

### 6.3 Handling Discrete-Time Biomolecular Omics Data

Most biomolecular omics data in bioinformatics are discrete in time, whereas existing graph-based dynamic anomaly detection algorithms typically target continuously evolving time-series data. Converting discrete data into continuous time-series data is therefore a major challenge for dynamic biomolecular network analysis [61]. Developing specialized mathematical models and computational methods for effective data transformation is a key focus for addressing this problem in the future.

## CONCLUSION

With the continuous development of science and technology and the ongoing exploration of human life information, the bioinformatics industry has experienced vigorous growth, leading to the emergence of various modern bioinformatics technologies and an increasing volume of biomedical omics data [62]. These data are often structurally complex, high-dimensional, and constantly evolving over time. In biomolecular networks, it is essential to focus not only on frequently occurring biomolecules with distinct patterns but also on abnormal molecules. Studying these abnormalities helps in understanding the occurrence and progression of complex diseases, revealing potential biological mechanisms, and providing clues for disease diagnosis and treatment. Therefore, the application of graph embedding-based anomaly detection algorithms in bioinformatics holds significant research and practical relevance.

This survey begins with a comprehensive introduction to the foundational knowledge of graph embedding and graph anomaly detection in bioinformatics. It reviews the development of graph embedding technologies, covering the research history and current state of graph embedding techniques based on random walks and neural networks. Classic algorithms and their improved variants are introduced, and current applications of graph embedding technology in bioinformatics are summarized. The general classification of anomaly detection algorithms and their specific applications in bioinformatics are then elaborated. Since most biomolecular networks are dynamically changing, this article further discusses research on dynamic anomaly detection algorithms based on graph embedding. Finally, the challenges and future directions for applying graph embedding-based anomaly detection algorithms in bioinformatics are examined. This review analyzes various deep learning models for graph embedding-based anomaly detection in bioinformatics and discusses the applicability of relevant technical methods, providing strong foundational support for future disease diagnosis, gene function research, and drug discovery.

**Expectation**

Although the continuous development of graph embedding technology has significantly advanced many bioinformatics tasks, these methods are generally designed for general graph data rather than specifically for biomedical omics data. As a result, their direct application in bioinformatics faces limitations and requires further exploration and optimization. Given the complex, heterogeneous, and high-dimensional nature of biomedical networks, developing graph embedding methods that effectively capture biological relationships while maintaining interpretability remains a critical challenge.

Moreover, the application of dynamic graph anomaly detection algorithms in bioinformatics is still in its infancy. Most existing studies focus on static graphs, overlooking the fact that biomolecular interactions and disease progression are inherently dynamic. Adapting anomaly detection techniques to dynamic graphs is essential for capturing temporal changes in biological systems, identifying disease evolution patterns, and improving early disease diagnosis.

Despite these challenges, the increasing integration of deep learning, artificial intelligence, and multi-omics data analysis provides exciting opportunities for advancing graph-based anomaly detection in bioinformatics. As research continues, we anticipate the emergence of more specialized methods tailored to biomedical applications. Based on these ongoing developments, this review will be continuously updated and expanded to reflect the latest advancements in the field.

## Figures and Tables

**Table 1:** Summary of graph embedding methods based on random walk and neural network.

**Category**	**Algorithm**	**Year**	**Time Complexity**	**Supports Dynamic Data**	**Figure Properties**		**Source code**
					**Homogeneous**	**Heterogeneous**	
Random walk	DeepWalk [17]	2014	*O*(*N* · *d*)	×	√	-	https://github.com/phanein/deepwalk
-	Node2vec [18]	2016	*O*(*N* · *d*)	×	√	-	https://github.com/aditya-grover/node2vec
-	WalkLets [19]	2017	*O*(*N* · *d*)	×	√	-	https://github.com/benedekrozemberczki/walklets
-	LINE [20]	2015	*O*(*E* · *d*)	√	√	-	https://github.com/tangjianpku/LINE
-	GraphWave [21]	2018	*O*(*N*^3^)	×	√	-	https://github.com/snap-stanford/graphwave
-	HARP [22]	2018	*O*(*N* log *N* + *N* · *d*)	×	√	-	https://github.com/GTmac/HARP
-	Metapath2vec [23]	2017	*O*(*N*_m_ · *d*)	×	-	√	https://ericdongyx.github.io/metapath2vec/m2v.html
-	Struc2vec [31]	2017	*O*(*N*^2^ + *N* · *d*)	×	√	-	https://github.com/leoribeiro/struc2vec
Neural network	GCN [26]	2016	*O*(*E* · *d* + *N* · *d*^2^)	√	√	√	https://github.com/tkipf/gcn
-	GAT [27]	2017	*O*(*E* · *d* + *N* · *d*^2^)	√	√	√	https://github.com/PetarV-/GAT
-	GraphSAGE [28]	2017	*O*(*N* · *S* · *d*)	√	√	-	https://github.com/williamleif/GraphSAGE
-	GAE [24]	2014	*O*(*E* · *d* + *N* · *d*^2^)	×	√	-	https://github.com/tkipf/gae
-	SDNE [29]	2016	*O*(*E* · *d* + *N* · *d*^2^)	×	√	-	https://github.com/suanrong/SDNE
-	Transformer [30]	2017	*O*(*N*^2^ · *d*)	√	√	√	https://github.com/huggingface/transformers

**Table 2:** Summary of the applications of graph embedding methods in bioinformatics.

**Data**	**Articles**	**Year**	**Embedding Algorithm**	**Tasks**
Omics data	Suratanee *et al.* [32]	2023	DeepWalk	Association identification
-	Zhu *et al.* [33]	2019	DeepWalk	Gene prioritization
-	Josifoski *et al.* [34]	2017	Node2vec	Function prediction
-	Zitnik *et al.* [35]	2017	GNN	Function prediction
-	Kang *et al.* [36]	2022	GCN	Association identification
-	Lu *et al.* [37]	2024	GraphSAGE	Association identification
-	Lagisetty *et al.* [38]	2022	GraphWave	Biomarker identification
Drug data	Ma *et al.*[39]	2018	GAE	Association identification
-	Chen *et al.* [40]	2020	DeepWalk	Association identification
-	Purkayastha *et al.* [41]	2019	Metapath2vec	Association identification
-	Hu *et al.* [42]	2020	GAT	Association identification
-	Liu *et al.* [43]	2023	SDNE	Association identification
-	Feng *et al.* [44]	2020	GCN	Association identification

**Table 3:** Summary of anomaly detection algorithms based on graph embedding.

**Algorithm**	**Year**	**Embedding Category**	**Measurement**	**Detection Method**	**Source Code**
DOMINANT [45]	2019	GCN; GAE	Anomaly Score	Graph structure	/
SpecAE [46]	-	GCN	Anomaly Score	Density; probability	/
AnomalyDAE [47]	2020	GAT; GAE	Anomaly Score	Graph structure	https://github.com/haoyfan/AnomalyDAE
DONE [48]	2020	GNN	Anomaly Score	Graph structure	https://bit.ly/35A2xHs
AANE [49]	2020	GCN; GAE	Anomaly probability	Graph structure	/

**Table 4:** Summary of anomaly detection algorithms based on dynamic graph embedding.

**Algorithm**	**Year**	**Embedding Category**	**Measurement**	**Source Code**
NetWalk [50]	2018	Random walk	Anomaly Score	https://github.com/chengw07/NetWalk
AddGraph [51]	2019	GCN	Anomaly Score	https://github.com/Ljiajie/Addgraph
TADDY [53]	2023	Transformer	Anomaly Score	https://github.com/yuetan031/TADDY_pytorch
GDN [54]	2021	GNN; GAT	Anomaly Score	https://github.com/d-ailin/GDN
StrGNN [52]	2020	GNN	Anomaly Score	https://github.com/LeiCaiwsu/StrGNN
H-VGRAE [55]	2020	GAE	Anomaly Score	/
DEGCN [56]	2022	GCN	Anomaly Prediction	/
HGCN [57]	2023	GCN	Anomaly Score	/
GLAD-PAW [58]	2021	GAT	Anomaly Prediction	/

## LIST OF ABBREVIATIONS

AANE: Anomaly Aware Network Embedding
ASD: Autism Spectrum Disorder
CBOW: Continuous Bag of Words
DEGCN: Dynamic Evolutionary Graph Convolutional Network
DTI: Drug-target Interactions
GAE: Graph Auto-Encoders
GAN: Generative Adversarial Networks
GAT: Graph Attention Networks
GCN: Graph Convolutional Networks
GDN: Graph Deviation Network
GMM: Gaussian Mixture Models
HGCN: Hierarchical Graph Convolutional Network
LINE: Large-scale Information Network Embedding
MGDHGS: Metabolite-gene-disease Heterogeneous Network
SDNE: Structural Deep Network Embedding
